# Multiple sexually transmitted co-infections are associated with adverse reproductive outcomes in asymptomatic adolescent pregnant women; A Prospective cohort study

**DOI:** 10.3389/fmed.2022.1046233

**Published:** 2022-11-17

**Authors:** Kirti Wasnik, Pratima Mittal, Priti Ghope, Subash C. Sonkar, Geetika Arora, Daman Saluja

**Affiliations:** ^1^Dr. B. R. Ambedkar Center for Biomedical Research, University of Delhi, New Delhi, India; ^2^Department of Obstetrics and Gynaecology, Vardhman Mahavir Medical College and Safdarjung Hospital, New Delhi, India; ^3^Delhi School of Public Health, Institute of Imminence, University of Delhi, New Delhi, India

**Keywords:** adolescent pregnancy, preterm birth (PTB), premature rupture of membranes (PROM), preterm premature rupture of membranes (PPROM), STI co-infections, adverse birth outcomes, asymptomatic adolescent pregnant women, pregnancy infections

## Abstract

**Background:**

A prospective cohort was conducted to assess the prevalence of seven RTIs/STIs in adolescent asymptomatic pregnant women to find a significant correlation between infection and pregnancy.

**Methods:**

The study was restricted to 18–19 years of asymptomatic adolescent pregnant women attending Ante-Natal Care and the health status of the pregnant women were followed up to parturition. The health status of the infant was followed till 6 months post-delivery. The prevalence of the concerning pathogens and the significance of their association with adverse outcomes of pregnancy were determined.

**Results:**

Among 279 subjects, the most significant co-infections were observed for *M. hominis with U. parvum* (9.31%; *p*-value–0.0071/OR−2.6421*)* and *U. urealyticum* (7.88%; *p*-value–0.0119/OR−2.6455). Statistically significant associations were found between *C. trachomatis* [(*p*-value-0.0439); OR−2.9902] and *M. genitalium* [(*p*-value−0.0284); OR−3.442] with PTB*, N. gonorrhoeae* with LBW <2.5 kg [(*p*-value−0.0052);OR−4.9017]*, U. urealyticum* with VLBW <2 kg [(*p*-value-0.0262);OR−3.0207]*, M. genitalium* [(*p*-value-0.0184); OR−11.7976] *and T. vaginalis with* PROM [(*p*-value 0.0063); OR−19.4275] *while M. genitalium* [(*p*-value 0.0190); OR**–**12.9230] *and U. urealyticum* [(*p*-value 0.0063); OR**-**14.5149] *with* PPROM with 95% CI respectively.

**Conclusions:**

Asymptomatic adolescents are at high risk of adverse pregnancy outcomes if infected with the concerned pathogens.

## Introduction

Reproductive tract infections (RTIs) are a major threat among sexually active women especially adolescent women due to high-risk sexual behavior, physiological susceptibility, and allelic variations/haplotypes of TNF-α toward the infections (1, 2). Adolescent pregnancy is the leading cause of adolescent maternal morbidity and mortality globally (3). Moreover, stillbirths are also prevalent among young adolescents in India (4), A high percentage of pregnant adolescents face complications like low birth weight (LBW), cesarean-section, and preterm deliveries (5). Our research group is working in the field of STI diagnostics for the past several years and observed that infections caused by *Chlamydia trachomatis* (CT) (6–9), *Neisseria gonorrhoeae* (NG) (9–14), *and Trichomonas vaginalis* (TV) (15–17) are most common sexually transmitted infections (STIs) with prevalence in the range of 7–29, 5–19 and 2–14% respectively, in symptomatic adult women (18). These pathogens may cause single infections or may coinfect (18–20).

A cross-sectional study reported, 49% women are asymptomatic for these infections (21). Univariate, studies showed *Mycoplasma genitalium* (MG) infection to be significantly associated with cervicitis (22); however, these infections are least studied in the Indian context. Although it was reported that *Mycoplasma hominis* (MH), *Ureaplasma urealyticum* (UU) and *Ureaplasma parvum* (UP) are part of normal vaginal flora, but their serovars responsible for the obstetrics complications (23) tend to increase antimicrobial resistance (24). It has been reported that *Ureaplasma* species are observed more frequently in the symptomatic patients, whereas *Mycoplasma* species are observed in asymptomatic pregnant women (25, 26).

Mothers infected with MH, TV, UU can transmit the infections to newborns (27). An association of CT with preterm birth (PTB) (28–30) and NG with LBW is reported (31, 32). The prevalence data for STIs are available in the context of the symptomatic reproductive age group (18–45). However, little is known about their effects in asymptomatic adolescents. These infections have been reported to contribute to infertility and adverse obstetric outcomes in symptomatic patients (23, 33). Adolescent physiologies impel them toward high-risk infections though negligible data exists with respect to adolescent pregnancy (34).

We therefore, decided to find prevalence of CT, NG, TV, MG, MH, UU and UP in asymptomatic adolescent pregnant women. Concerned women visiting the Safdarjung hospital, India during 2014–2015 were recruited after taking their consent as per the ethical guidelines. Samples were collected during the first trimester and used for testing the presence of CT, NG, TV and genital mycoplasmas (MH, MG, UU, UP). We analyzed the percentage of maternal and fetal adverse outcome observed in asymptomatic pregnant adolescents and tried to establish correlations between adverse sequelae and pathogenic infections/co-infections in the mothers.

## Methods

### Study design and population

**Figure 1:**
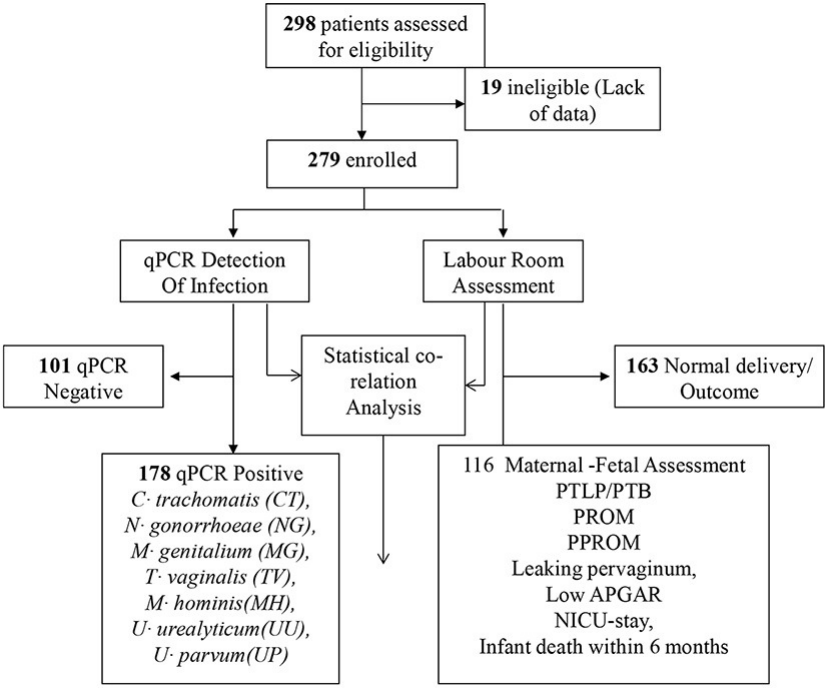
Prospective cohort study layout.

Prospective cohort study, conducted in department of obstetrics and gynecology, VMMC and Safdarjung hospital, New Delhi, India in collaboration with Dr. B R Ambedkar center for biomedical research (Figure 1). Assuming the minimum prevalence of Chlamydia, Neisseria, genital Mycoplasmas, and trichomoniasis infections in asymptomatic adolescent pregnant women to be 5%, 0.05 alphas, and 80% power, 298 subjects were recruited. The study was restricted to 18–19 years of asymptomatic adolescent pregnant women attending Ante-Natal Care (ANC) OPD during the period of 2014 to 2015, irrespective of gestation and parity. Subjects with diabetes mellitus, hypertension, tuberculosis, severe anemia, were excluded from the study. Those who had symptomatic Urinary Tract Infections with organisms other than STD, human immunodeficiency Virus positive, gone through multiple pregnancies were also excluded. Enrolled women who did not receive antenatal care throughout gestation or did not undergo parturition at Safdarjung hospital were also excluded from the study.

### Data collection

All the recruited subjects with complete data profile, between the age group of 18–19 years and visiting the Obstetrics and Gynecology department OPD of the hospital for their first pregnancies were clinically examined. After confirmation of pregnancy, the health status of women was followed-up till parturition. Moreover, the health status of infants was followed up to age of 6 months *via* telephonic conversations. During parturition, maternal and fetal outcomes were also assessed and documented. The following maternal parameters; 1) Time and period of gestation at delivery term/preterm, 2) preterm labor pain, 3) preterm premature rupture of amniotic membrane (spontaneous rupture of membrane before 37 weeks of gestation and without the presence of labor pain), 4) premature rupture of membrane (spontaneous rupture of the membrane after 37 weeks of gestation before the onset of labor), 5) Chorioamnionitis, 6) mode of delivery, 7) sign and symptoms of postpartum endometritis (fever, foul smell and vaginal discharge) were studied. We also studied the following fetal parameters; 1) APGAR Score (1–5 min), 2) Low Birthweight (birth weight < 2.5 kg), 3) neonatal Intensive care unit (NICU) stay and 4) Signs and symptoms of pneumonia and ophthalmia neonatorum.

### Specimen collection and infection diagnosis

Cervical swab samples were collected from 298 subjects and stored at −20°C until used. Collected samples were screened for *Chlamydia trachomatis, Neisseria gonorrhoeae, Mycoplasma genitalium, Mycoplasma hominis, Ureaplasma urealyticum, Ureaplasma parvum and Trichomonas vaginalis*. Genomic DNA was isolated from each specimen using Macherey-Nagel™ NucleoSpin™ Tissue kit, followed by a quantitative PCR assay using FTD Urethritis Plus kit (CE 0123) as per manufacturer's instructions.

### Statistical analysis

Data are presented using descriptive statistics using Meta Chart software (https://www.meta-chart.com/venn). Comparison of data between groups was done using χ^2^ tests or univariate conditional logistic regression test. Statistical analysis was conducted using MedCalc software (https://www.medcalc.org/) or Prism GraphPad online tool (https://www.graphpad.com/quickcalcs/contingency1/). A *p*-value <0.05 was considered statistically significant.

## Results

The study was designed to estimate the burden of reproductive tract infections (RTIs) among asymptomatic adolescent pregnant girls. Further, the goal was to estimate the association of prior infection or infection during the early weeks of pregnancy and the development of adverse events that affect health of mother and infant.

### Evaluation for the presence of seven RTI causing pathogens

**Figure 2:**
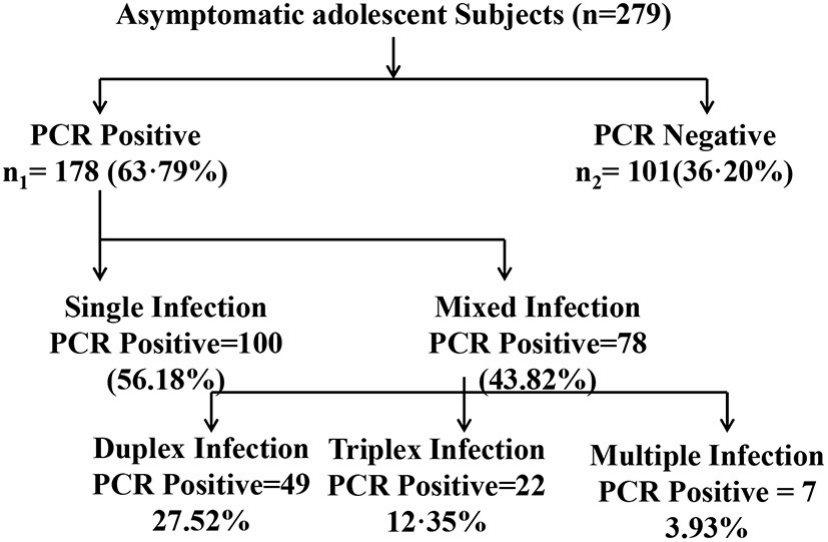
Diagrammatic representation of distribution of infected and noninfected subjects for single or mixed infection. The distribution of infection with CT, Chlamydia trachomatis; NG, Neisseria gonorrhoeae; MG, Mycoplasma genitalium; TV, Trichomonas vaginalis; MH, Mycoplasma hominis; UU, Ureaplasma urealyticum; UP, Ureaplasma parvum out of the total asymptomatic adolescent subjects enrolled in the study is shown.

During the period of 2014 to 2015, a total of 279 asymptomatic adolescent pregnant women visited the OPD of obstetrics & gynecology department of Safdarjung hospital with regards to their first conceivement. Cervical specimens were collected and tested for the presence of CT, NG, MG, MH, TV, UU and/or UP infection. Subsequently, 178/279 (63.21%) subjects tested positive for the presence of at least one of the concerned pathogens. Remaining 101/279 (36.78%) subjects were tested negative for these infections. Amongst the 178 positive subjects, 100 (56.18%) subjects had single infection while 78 (43.82%) subjects were co-infected with more than one pathogen. Further, 58.97% (46/78 subjects) subjects were co-infected with two pathogens, 28.20% (22/78 subjects) were co-infected with three pathogens and 12.82% (10/78 subjects) were found out to be positive for the presence of more than 3 tested pathogens (Figure 2).

**Figure 3:**
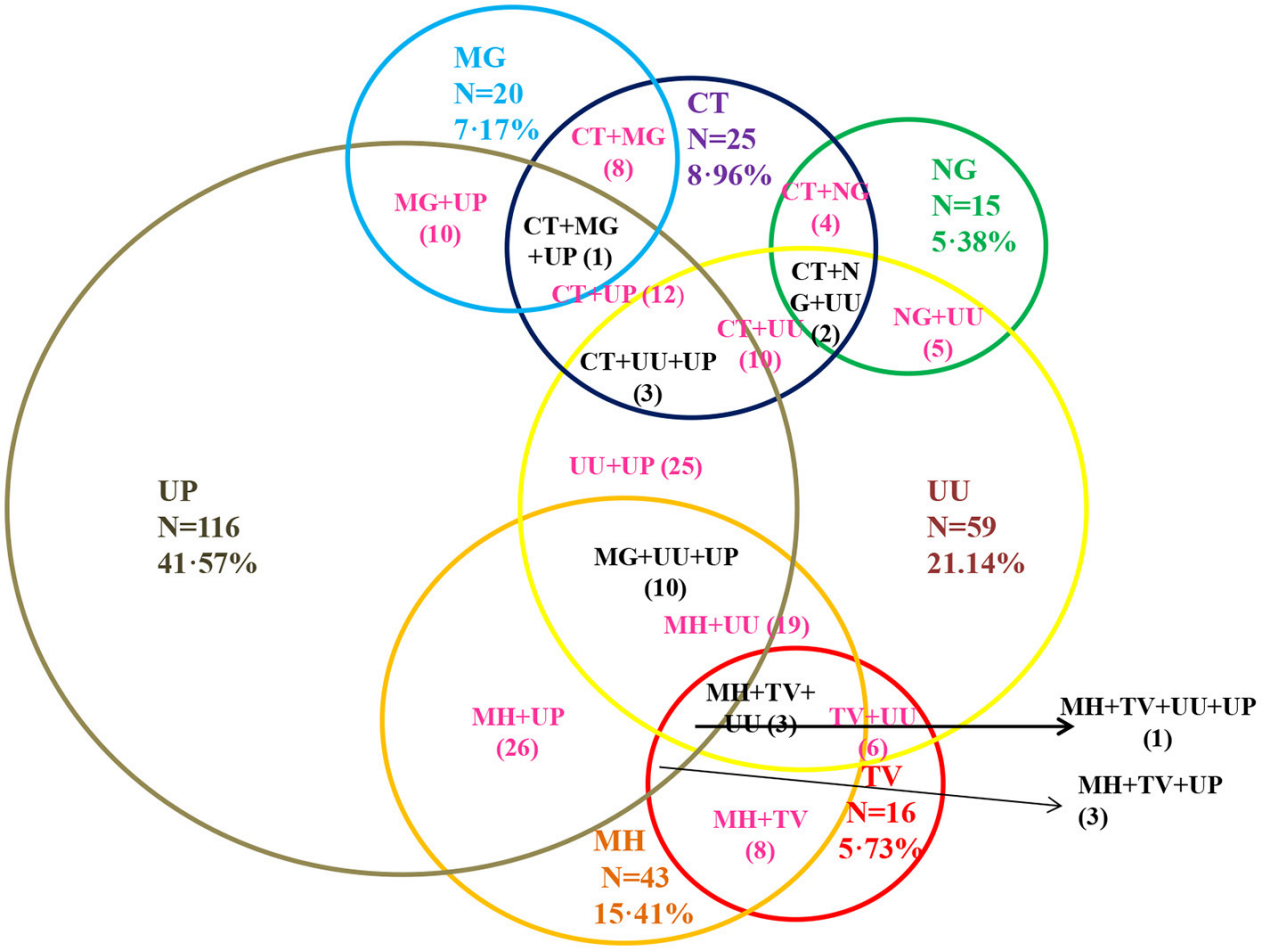
Prevalence of sexually transmitted infection and genital mycoplasmas among the asymptomatic adolescent girls. CT, Chlamydia trachomatis; NG, Neisseria gonorrhoeae; MG, Mycoplasma genitalium; TV, Trichomonas vaginalis; MH, Mycoplasma hominis; UU, Ureaplasma urealyticum; UP, Ureaplasma parvum out of the total asymptomatic adolescent subjects enrolled in the study is shown. DNA was isolated from samples collected from asymptomatic adolescent women and infection for these seven pathogens was determined by Real time PCR based diagnosis. Among total positive prevalence for CT, NG, MG, MH,TV, UU, UP were 8.96, 5.38, 7.17, 15.41, 5.73, 20.79, and 41.22%, respectively.

Among the 279 asymptomatic adolescent pregnant women, 25 (8.96%), 15 (5.38%), 20 (7.17 %), 43 (15.41%), 16 (5.73%), 59 (21.14%) and 116 (41.57%) were tested positive for the presence of CT, NG, MG, MH, TV, UU and UP respectively (Figure 3). Estimation of risk of co-infections.

**Figure 4:**
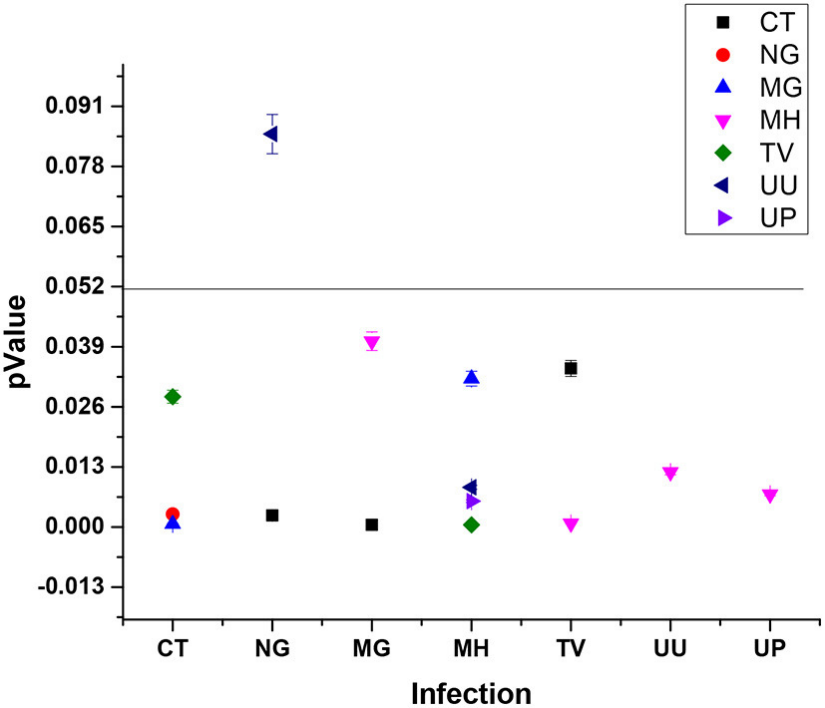
Pathogen significantly causes the co-infection inter-relationship (type 1 pathogen increase the risk of infection with other pathogen).

Most prevalent and significant co-infection was analyzed through logistic regression correlation test (Figure 4, Table 1). Among the 25 samples that were PCR positive for the presence of CT, 23 subjects were co-infected with one or more tested pathogens. CT infection was found to significantly increase the risk of co-infection with NG, MG and TV with *p*-values of 0.0028, 0.0007, and 0.0282 respectively (Figure 4) and with odds ratio 6.9949 (1.9495 to 25.80974), 7.3984 (2.3247 to 23.5453) and 4.311 (1.1687 to 15.9028) respectively with 95% CI (Table 1). MG infections have been found to be significantly associated with CT and MH with a *p*-value of 0.0005, 0.0402; OR value of 7.7628 (2.4332 to 24.7659; CI 95%) and 0.088(0.0086 to 0.8975; CI 95%) respectively. MH infection was more frequently evident with other pathogens than CT and NG. MH infection was significantly associated with MG (*p*-value 0.0321), TV (*p*-value 0.0005), UU (*p*-value 0.0086) and UP with (*p*-value 0.0056) and odds ratios 0.0771 (0.0074 to 0.8033), 8.6516(2.5846 to 28.9598), 2.7836 (1.2966 to 5.9762) and 2.758 (1.3456 to 5.6529) respectively. TV was frequently spotted with MH (*p*-value 0.0008) and CT (*p*-value 0.0343) with respective OR 8.2457 (2.3932 to 28.4106) and 4.2788 (1.1136 to 16.4405). UU and UP were found to increase the risk of infection of MH/UU, or MH/UP co-infection with *p*-value of 0.0119, 0.0071 and OR 2.6455 (1.2391 to 5.6482) and 2.6421 (1.3024 to 5.3596) respectively (Table 1).

**Table 1:** Statistical association for risk for co-infection of pathogen.

***p*-value/Odd Ratio; 95%CI**	**CT**	**NG**	**MG**	**MH**	**TV**	**UU**	**UP**
CT		0.0028/6.9949;1.9495 to 25.80974	0.0007/ 7.3984; 2.3247 to 23.5453	0.137/ 2.3066; 0.7666 to 6.9401	0.0282/ 4.311; 1.1687 to 15.9028	0.8112/ 1.1326; 0.4076 to 3.1469	0.9594/ 1.0239; 0.4123 to 2.5426
NG	0.0025/ 7.1781; 2.0022 to 25.7344		0.7783/ 1.2883; 0.2729 to 5.3355	0.8044/ 1.2066; 0.2729 to 5.3355	0.9976/ 2.58E-09	0.085/ 2.7711; 0.8688 to 8.8386	0.887/ 0.9208; 0.2949 to 2.8750
MG	0.0005/ 7.7628; 2.4332 to 24.7659	0.8083/ 1.2413; 0.2164 to 7.1190		0.0402/ 0.088; 0.0086 to 0.8975	0.1303/ 3.674; 0.6807 to 19.8306	0.3076/ 1.7883; 0.5854 to 5.4633	0.2306/1.8218; 0.6832 to 4.8574
MH	0.1759/ 2.1699; 0.7068 to 6.6617	0.7548/ 1.2544; 0.3025 to 5.2010	0.0321/ 0.0771; 0.0074 to 0.8033		0.0005/ 8.6516; 2.5846 to 28.9598	0.0086/ 2.7836; 1.2966 to 5.9762	0.0056/ 2.758; 1.3456 to 5.6529
TV	0.0343/ 4.2788; 1.1136 to 16.4405	0.9976/ 3.01E-09	0.1017/ 3.9327; 0.7631 to 20.2681	0.0008/ 8.2457; 2.3932 to 28.4206		0.5461/ 1.4443; 0.4378 to 4.7642	0.2154/ 0.4772; 0.1480 to 1.5383
UU	0.7609/ 1.1696; 0.4264 to 3.2086	0.1037/ 2.5711; 0.8244 to 8.0182	0.3196/ 1.7298; 0.5879 to 5.0896	0.0119/ 2.6455; 1.2391 to 5.6482	0.3914/ 1.6544; 0.5232 to 5.2313		0.7139/ 0.8913; 0.4817 to 1.6491
UP	0.985/ 0.9913; 0.3002 to 2.4622	0.7883/ 0.8579; 0.2803 to 2.6259	0.2394/ 1.7868; 0.6793 to 4.6995	0.0071/ 2.6421; 1.3024 to 5.3596	0.301/ 0.5504; 0.1775 to 1.7065	0.6923/ 0.8829; 0.4756 to 1.6358	

### Asymptomatic adolescent pregnancy outcome

**Figure 5:**
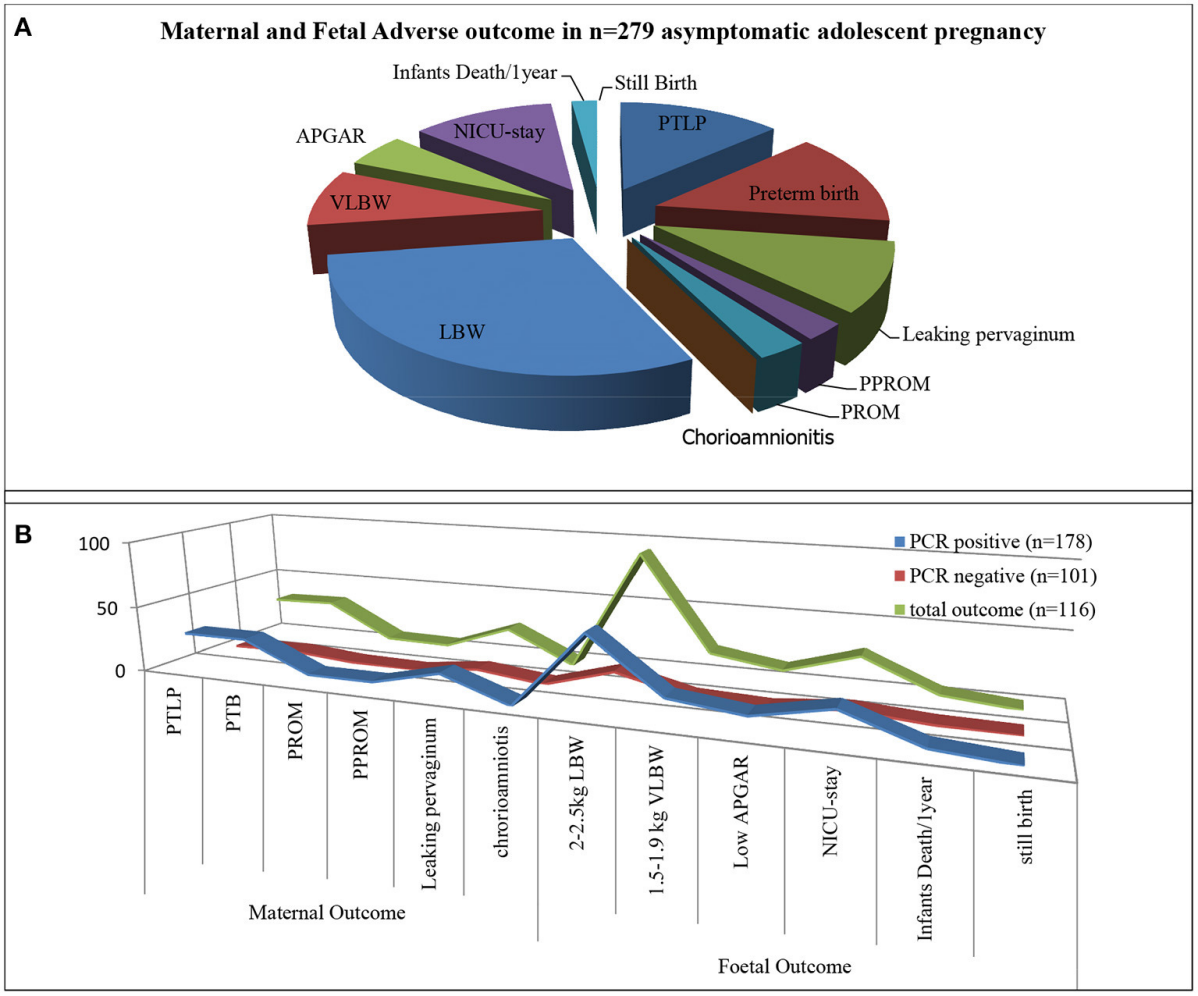
Infection and maternal and fetal outcome. **(A)** Total adverse pregnancy outcome observed in asymptomatic adolescent. **(B)** Represent adverse pregnancy outcome observed in PCR tested positive group (blue strip), PCR tested Negative group (Red strip) from total adverse outcome (green strip).

Total 279 subjects were assisted from the beginning of pregnancy till the delivery. Maternal-fetal adverse outcomes were observed in 116/279 (41.57%) subjects whereas, 163/279 (58.42%) had normal maternal-fetal outcomes (Figure 1). 33/279 (11.82%) subjects exhibited PTB, 32/279 (11.46%) showed PTLP, 6/279 (2.15%) subjects showed PPROM, 7/279(2.51%) subjects had PROM, 24/279(8.60%) subjects reported leaking per vaginum and 92/279 (32.97%) subjects gave birth to a fetus with low birth weight (below 2.5 kg). Amongst 92/279 (32.97%) fetuses, 72/116 (62.06%) had a birth weight of 2.1–2.5 kg while 20/116 (17.24%) had below 2 kg birth weight. 12/279(4.30%) fetuses showed low APGAR value (below 5), 28/279 (10.03%) infants needed NICU stay care and 6(2.15%) infants died within 1 year from birth. Among the study population, none of the subjects developed chorioamnionitis, postpartum endometritis and stillbirth (Figure 5A).

### Infections increase the risk of adverse pregnancy outcomes: Statistical correlation

**Figure 6:**
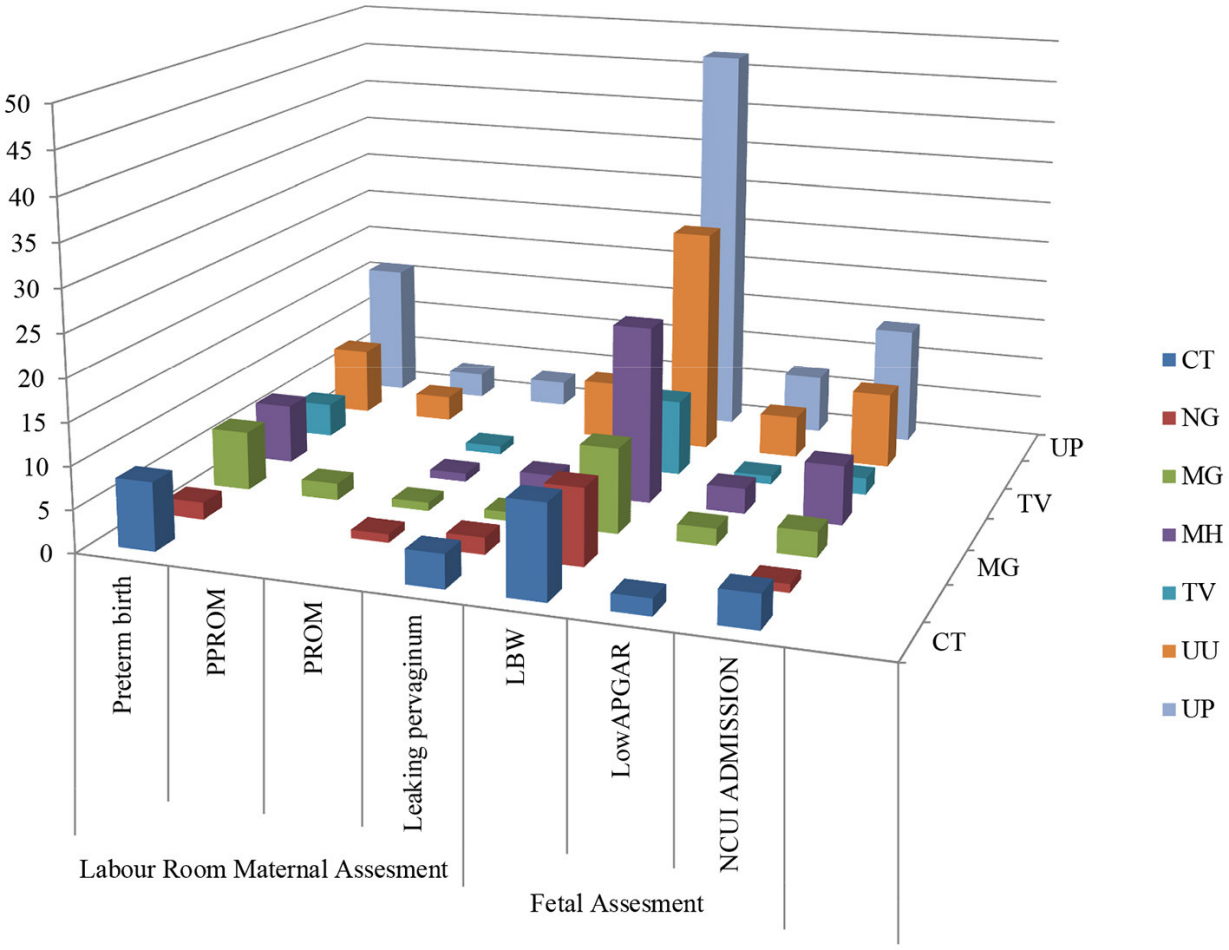
Association between infection/ co-infection and maternal and natal related adverse events.

Adverse sequels were categorized in maternal outcomes *i.e.*, PROM, PPROM, chorioamnionitis, PTLP, PTB and natal related outcome LBW, low APGAR value and NICU admission. Very few subjects were observed with PROM and PPROM while none of the subjects were observed with chorioamnionitis. Statistical correlation between infections and adverse outcome were analyzed by applying the χ^2^ tests. Our results indicate that 178 subjects were PCR positive for at least one pathogen and 101 subjects were negative. High distribution of adverse outcomes was observed in PCR positive subjects than that in PCR negative subjects (represented in Figure 5B). Ninety subjects are true positive and 75 subjects are true negative for infections and adverse outcomes. Eighty eight subjects tested positive by PCR but did not show adverse outcomes while 26 subjects were negative for infection but showed adverse outcomes (Table 2). These results show statistically significant correlation between maternal/fetal adverse outcomes and infections, with a *p*-value of 0.0009 (*p* < 0.05) with yates' correction 15.3351. The pattern of distribution of infection related to adverse sequel is represented in (Figure 6, Table 2). Except CT and UU, all pathogens were observed in various subjects who developed PROM, while only MG, UU and UP pathogens were found to be associated with subjects who developed PPROM. With respect to the fetus with low APGAR value, none of the mothers was diagnosed with NG infection. While all the other pathogens were significantly associated with low APGAR values in neonates as shown in Figure 6.

**Table 2:** Correlation between maternal and fetal adverse outcome and infection.

**Infection**	**Adverse outcome positive**	**Adverse outcome negative**	**Total**	***p*-value significance p < 0.05**
PCR Positive	90	88	178	Significant
PCR Negative	26	75	101	*p*-value = 0.00009
Total	116	163	279	yate's correction = 15.3351

**Figure 7:**
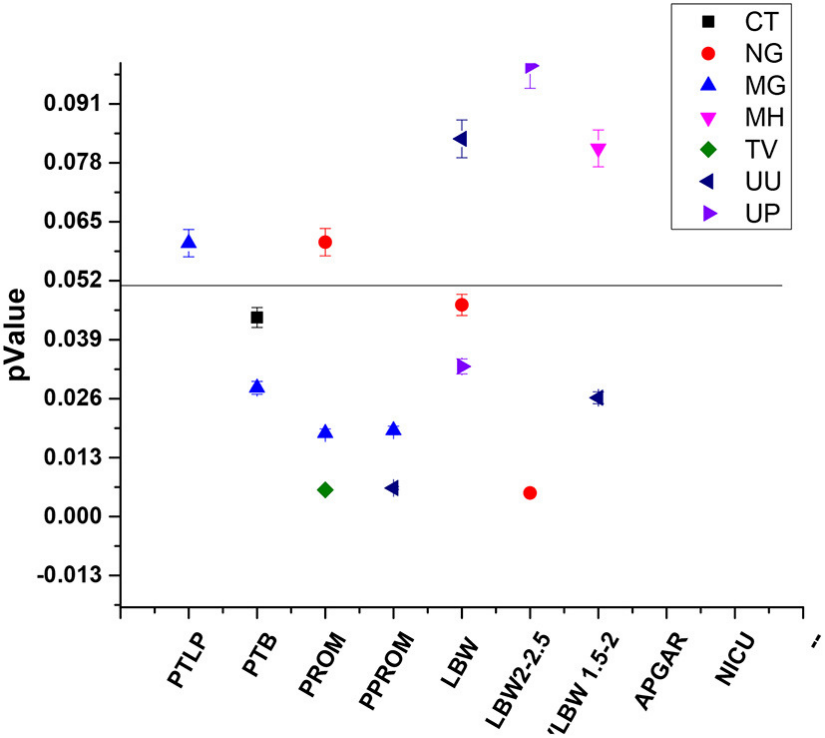
Statistically significant association between STIs/RTIs with maternal and fetal adverse outcomes.

Our study majorly focused on asymptomatic infections in adolescent subjects and their association for increasing the risk of adverse outcomes, therefore logistic regression statistical analysis was applied to all the 279 subjects' variable data sets. Association of infection and its high-risk behavior among the adolescent and infants were statistically validated at *p*-value of 0.05 and logistic regression represented in Figure 7, Table 3.

**Table 3:** Statistical association between type of infection and adverse outcomes.

***p*-value/Logistic regression; Odd ratio (95%CI)**	**PTB**	**PTLP**	**PROM**	**PPROM**	**Birth weight**			**APGAR below 5**	**NICU admission**
					**LBW**	**LBW 2.5 kg**	**VLBW 1.5 kg**		
CT	**[**0.0439**];** 2.9902 **(**1.0305 to 8.6767**)**	[0.1064]; 2.4395 (0.8264 to 7.2015)	0.9976	[/0.9986]; 1.26E-09	[0.8723];0.9254 (0.3592 to 2.3838)	[0.7339]; 1.1816 (0.4516 to 3.0917)	[0.2719]; 0.2897 (0.0318 to 2.6413)	[0.49]; 1.8569 (0.3203 to 10.7642)	[0.4408]; 1.6458 (0.4637 to 5.8413)
NG	[0.5971]; 1.4792 (0.3464 to 6.3161)	[0.9551]; 0.9542 [0.1867 to 4.8780]	[0.0605]; 10.6384 (0.9006 to 125.6664)	[/0.9989]; 8.94E-10	[0.0467]; 3.1312 (1.0167 to 9.6438)	**[**0.0052**];** 4.9017 **(**1.6056 to 14.9644**)**	[0.9878]; 1.0169 (0.1181 to 8.7563)	[0.9981]; 5.62E-09	[0.4771]; 0.4616 (0.0548 to 3.8889
MG	**[**0.0284**];** 3.4422 **(**1.1395 to 10.3982)	[0.0603]; 2.9889 (0.9538 to 9.3656)	**[**0.0184**];** 11.7976 **(**1.5158 to 91.8199**)**	**[**0.0190**];** 12.9230 **(**1.5218 to 109.7444**)**	[0.2018]; 1.9224 (0.7047 to 5.2441)	[0.2739]; 1.7673 (0.6372 to 4.9018)	[0.5821]; 1.6116 (0.2945 to 8.8180)	[0.3487]; 2.3077 (0.4014 to 13.2664)	[0.5606]; 1.5097 (0.3770 to 6.0455)
MH	[0.8779] 0.9161 (0.2996 to 2.80173.0321)	[0.4428]; 1.4911 (0.5375 to 4.1362)	[0.6547]; 0.5897 (0.0512 to 6.7956)	[0.9982]; 1.52E-09	[0.1652]; 1.6681 (0.8098 to 3.4361)	[0.7372]; 1.1426 (0.5245 to 2.4892)	[0.0812]; 2.6010 (0.8881 to 7.6174)	[0.6793]; 1.3727 (0.3057 to 6.1643)	[0.3021];1.7069 (0.6183 to 4.7120)
TV	[0.8843]; 1.1199 (0.2437 to 5.1467)	[0.4222]; 1.7473 (0.4472 to 6.8268)	**[**0.0059**];** 19.4275 **(**2.3163 to 162.9430)	[0.9988]; 1.04E-09	[0.1456]; 2.2852 (0.7509 to 6.9549)	[0.1528]; 2.2607(0.7390 to 6.9152)	[0.6217] 1.5401; (0.2770 to 8.5625)	[0.7744]; (0.0676 to 7.4423	[0.7407]; 0.7529 (0.1402 to 4.0428)
UU	[0.9180]; 1.0494 (0.4191 to 2.6277)	[0.7067]; 0.8309 (0.3167 to 2.1804)	[0.3222]0.2632; (0.0187 to 3.6994)	**[**0.0063**];** 14.5149 **(**2.1289 to 98.9616**)**	[0.0833]; 1.7411 (0.9295 to 3.2613)	[0.4748]; 1.2753 (0.6546 to 2.4845)	**[**0.0262**];** 3.0207 **(**1.1399 to 8.0049**)**	[0.104]; 2.8317 (0.8074 to 9.9311	[0.1968]; 1.7987 (0.7376 to 4.3863)
UP	[0.6511];1.1938 (0.5540 to 2.5724)	[0.392]; 1.4017 (0.6470 to 3.0365)	(0.6010) 1.5527 (0.2985 to 8.0757)	[0.8632]; 1.1689 (0.1980 to 6.8987)	[0.0331]; 1.7808 (1.0473 to 3.0280)	[0.0994]; 1.6081 (0.9139 to 2.8296)	[0.1738]; 1.9624 (0.7427 to 5.1850)	[0.3213]; 1.8565 (0.5466 to 6.3059)	[0.4673]; 1.3501 (0.6010 to 3.0328)

#### Adolescent asymptomatic infections in pregnancy lead to preterm birth

32 and 33 subjects developed PTLP and gave birth preterm (PTB) respectively. Amongst these 28 subjects tested positive for at least one pathogen, while 4 subjects tested negative for any infection developed PTLP and 5 subjects developed PTB. None of the pathogens under study could be significantly correlated to PTLP while CT and MG infections show good correlation in increasing the chances of PTB With *p*-values 0.0439 and 0.0284, and odds ratios 2.9902 (1.0305 to 8.6767 95% CI) and 3.4422 (1.1395 to 10.3982 95% CI) respectively, at 95% CI (Figure 7, Table 3).

#### Asymptomatic adolescent infections associated with PROM

Only 7 subjects out of 279 (2.50%) developed adverse event PROM. From 178 PCR positive subjects, only six subjects developed PROM and were infected with either single, or co- infection of NG, TV, MH, UU and UP. On the other hand, from 101 PCR negative subjects, only one subject developed PROM. Although the total number of subjects were low, we observed strong co-relation of PROM with TV and MG infections with *p*-value 0.063 [odds ratio 19.4275 **(**2.3163 to 162.9430; 95% CI)] and 0.018 [11.7976 **(**1.5158 to 91.8199; 95%CI)] respectively (Figure 7, Table 3).

#### Asymptomatic adolescent infections associated with PPROM

PPROM was observed in 6/279 (2.15%) subjects and all six subjects tested positive for MG, UU and UP co-infection (Figure 7). Similar to PROM, we observed strong correlation of PPROM with MG infection (*p*-value 0.0190 and coefficient 2.86678) and then with UU infection (*p*-value 0.0250 and coefficient 2.11849) but not with TV (0.9988) (Figure 7). Nevertheless, the data is suggestive of effect of MG and UU infection on preterm premature rupture of membrane with OR 17.5802 (2.0676 to 149.4820); 95% CI) and 8.3186 (1.3042 to 53.0590; 95%CI) respectively (Table 3).

#### Adolescent infection and risk of low birth weight of fetus

92 out of 279 subjects delivered infants with low birth weight, from those 72/92 subjects gave birth to infants with weight between 2.1 kg to 2.5 kg While 20/92 infants had the birth weight below 2 kg. From the 178 PCR positive tested mothers for one or more infections, birth weights of 57 infants were between 2.1–2.5 kg and 18 infants were born with weight below 2 kg. While from 101 PCR negative group, 15 infants had birth weight 2.1–2.5 kg and Only two infant's birth weight was below 2 kg. Thus, significant association was observed between infected mother and LBW of fetus. Overall, NG and UP infections significantly correlated with increased risk of low birth weight of fetus with *p*-values [(0.0467); OR 3.1312 (1.0167 to 9.6438)] and [(0.0331); 1.7808 (1.0473 to 3.0280)] Respectively, with respective coefficients of 1.14142 and 0.57704. We observed that NG infection was significantly associated with low birth weight (below 2.5 kg) Outcome with 0.0052 *p*-value and OR 4.9017 **(**1.6056 to 14.9644 at 95% CI**)**. While subjects infected with UU gave birth to VLBW infants with weight below 2 kg [*p*-value 0.0262; or 3.0207 (1.1399 to 8.0049 at 95% CI)] (Table 3). Our study also showed the subjects who had preterm delivery, gave birth to infants with low birth weight with OR 18.4026 (5.1966 to 65.1686; 95% CI) (Table 3).

#### Adolescent infections and other outcomes

Leaking per vaginum was observed in 8.96% asymptomatic pregnant women. We didn't observe any association with leading infection. Total 12/279 (4.3%) infants showed low APGAR score <5 while 28/279 (10.35) had to be admitted in NICU. These included all the 12 infants who had low APGAR score. Mothers of all these 12 infants tested positive for the infections. Similarly, with respect to 178 PCR positive mothers, infants of 23 were admitted in NICU and concerning 101 PCR negative mothers, only infants of 5 among them were admitted in NICU. However, no significant association was observed with concerned RTIs/STIs. Subsequently, 1.79% infant deaths were observed and among them, 0.71% died within 1 month while 1.07% died within a year without any significant association with any of these infections in mother.

Thus, among the 178 PCR positive subjects, high percentage were found associated with adverse outcomes which are as follows: PTLP-15.73% (28/178), PTB- 15.73% (28/178), PROM- 3.37% (6/178), PPROM 3.37% (6/178), leaking per vaginum 10.11% (18/178), LBW infants 32.02% (57/178) and VLBW 10.11% (18/178), Low APGAR 6.74% (12/178). However, 101 subjects who were PCR negative for these pathogens, also developed adverse outcomes but to a lower percentage. These include PTLP 3.96% (4/101), PTB 4.95% (5/101), PROM 0.99% (1/101), PPROM 0, leaking per vaginum 5.94% (6/101), LBW infants 14.85% (15/101) and VLBW 1.98% (2/101) (Supplementary Table S2).

## Discussion

Sexually transmitted infections (STIs) have long been associated with various adverse outcomes of pregnancy. These include sequelae in mothers ranging from PROM, PPROM to PTB as well. Some pathogens have also been reported to cause postpartum endometritis in women (32, 35, 36). On the other hand, they have been indicted to be responsible for low birth weight of fetus, ophthalmia neonatorum, low APGAR and also still-births in extreme cases (37). This study was aimed to determine the sequelae of pregnancy in adolescent women as a consequence of presence of CT, NG, TV, MG, MH, UU and/or UP in pregnant mothers, tested in the first trimester.

### Main findings

In the present study the percentage of infections were observed to be 7.17, 15.41, 20.79, 41.22 8.96, 5.38 and 5.73% for MG, MH, UU, UP, CT, NG and TV respectively among north Indian asymptomatic adolescent pregnant women. Nearly 43.82% subjects out of 178 infection positive samples, were co-infected for two or more pathogens. Among these, significant numbers of co-infections were found between CT and NG, CT and MG, CT and TV, MG and MH, TV and MH, MH and UU, and MH and UP were highly prevalent.

Highest percentage of infection was observed for Ureaplasma infection which significantly increases the risk for delivery of LBW infants. Koch *et al*. reported that CT or NG infection/co-infection raised the incidence of MH (33). Interestingly we observed that CT infected patients were more frequently co-infected with NG than with MG and TV but not significantly associated with MH (Table 1). Earlier it was reported that trichomoniasis increased the colonization with MH, Similarly, in this study group MH infection was more commonly observed with MG, TV, UU, UP with highest risk for TV co-infection and vice versa. About 41.57% subjects from total 279 subjects had been diagnosed with UP silent infection and 21.14% with UU infections which significantly increases the risk for MH infection.

Obstetric/fetal outcomes include PTB, PROM etc. and among the total asymptomatic adolescents 41.57% subjects developed complications. However, we did not observe any case of stillbirth or chorioamnionitis. Infants born to teenage mothers aged 17 or younger are reported to have higher risk for low Apgar score at 5 min (34) and these fetus need NICU care.

### Strengths

Though the previous studies are inconsistent, RTIs are considered to be one of the major causes for obstetric adverse sequel. This is the first study wherein association of multiple RTIs with obstetric adverse outcomes has been studied in asymptomatic adolescent pregnancy. By applying the chi-square statistics, our results showed significant co-relation between PCR tested positive infections and adverse outcomes. As our sample set includes asymptomatic subjects, we didn't distribute the population in control and test group. And for this variable data set, statistical significance is evaluated through logistic regression analysis. As we had collected the samples for PCR diagnosis only in first trimester, we hypothesize that development of adverse outcomes in PCR negative subjects may be due to infection that occurred later during pregnancy.

In antenatal period, there is an increased risk of miscarriage as well as PTLP during labor. There is increased risk for premature birth, premature rupture of membrane, chorioamnionitis and low fetal birth weight (38). We did not observe any association with PTLP but observed significant association of PTB with RTIs/STIs.

Chlamydial infection and its adverse outcomes have been studied extensively, however reports regarding obstetric outcome are inconsistent. Reekie et al. conducted a large cohort-based study and found no significant association of CT with PTB (30). However, in the present study, we found significant association of CT and MG with PTB in adolescent women. Moreover, preterm birth significantly increased the risk for low-birth weight infant. Our findings support the findings of Rours *et al*. wherein it is reported that CT infection is associated with PTB but not with other sequels (28)^.^ Olson-chce *et al*. reported association of CT with PTB as well as with LBW, endometritis PROM and PPROM (29) but we have not observed any such association.

Another most common STI causing pathogen is NG, which is often observed in co-infection with CT. As reported earlier, we also observed that maternal gonorrhea is significantly associated with LBW of fetus (31). Further, we also report significant association of UP with LBW of fetus which could be due to the fact that UP colonization is known to cause premature delivery (39). Higher detection of Mycoplasma and Ureaplasma significantly lower gestational age and correspondingly lower birth weight (38). UU infection leads to very low birth weight adverse sequel. Similar to these reports, we also observed significant effect of UU infection with PROM (OR 8.3186 at 95% CI). However, we did not find any correlation between MH infection and obstetric outcome in asymptomatic pregnancy. MH in cervical fluid has been reported to be associated with high risk of microbial invasion into amniotic cavity and histological chorioamnionitis together during pregnancy which is further complicated by PPROM (40) but we didn't find any subject with chorioamnionitis and still-birth. Some studies have exhibited TV infection to be associated with LBW, PTB and PROM (41). However, present study shows TV infection to be significantly associated with only PROM with highest OR 19.9972 (2.3735 to 168.4781).

Non-gonococcal urethritis caused by MG and MH is mostly associated with persistent and recurrent urethritis (42). MG related adverse obstetric outcome remain un-established but its presence strongly correlates with disease because its detection rate is rare among healthy individual. In the present study, we found that MG infection was significantly associated with PTB, PROM, and PPROM as well.

### Limitation

This Cohort study can be extended further as this study only focused toward the asymptomatic adolescents. In the present study we got very low number of sample for PROM, and PPROM.

### Interpretation

Adolescent women are at higher risk of encountering adverse pregnancy outcomes if infected by one or more of the STI causing pathogens considered in the study.

## Conclusion

All the pathogen except MH have significant association with obstetric adverse outcome in asymptomatic adolescents. CT and MG infections significantly increase the risk of Preterm birth, MG and TV cause the PROM and MG and UU infection increase the incidence of PPROM. NG infection significantly increases the risk of LBW infants while UU infection significantly increases the risk of VLBW. Hence, adolescents are at high risk and we recommend for testing of these pathogen in pregnancy especially for MG.

## Data availability statement

The original contributions presented in the study are included in the article/Supplementary material, further inquiries can be directed to the corresponding author.

## Ethics statement

The studies involving human participants were reviewed and approved by ICMR guidelines IEC/VHHC/SJH/THESIS/OCT-12 dated 5/5/2014. The patients/participants provided their written informed consent to participate in this study.

## Author contributions

KW, PG, PM, SCS, and DS conceptualized the study. KW, SCS, and GA carried out the experiments and data analysis. Collection of samples, maternal and fetal assessment, and data collection was carried out by PG and PM. KW, PM, SCS, and DS wrote and finalized the manuscript. All authors had full access and take full responsibility for data collection, data analysis, and final responsibility for the decision to submit for publication. All authors read and approved the final manuscript.

## Conflict of interest

The authors declare that the research was conducted in the absence of any commercial or financial relationships that could be construed as a potential conflict of interest.

## Publisher's note

All claims expressed in this article are solely those of the authors and do not necessarily represent those of their affiliated organizations, or those of the publisher, the editors and the reviewers. Any product that may be evaluated in this article, or claim that may be made by its manufacturer, is not guaranteed or endorsed by the publisher.
