# Supplementary material for: Multiple sexually transmitted co-infections are associated with adverse reproductive outcomes in asymptomatic adolescent pregnant women; A Prospective cohort study
Source: Front Med (Lausanne). 2022 Nov 17;9:1046233. doi: 10.3389/fmed.2022.1046233 (PMC9714506; doi:10.3389/fmed.2022.1046233)
Supplement: Supplementary file 1 [file Table_1.DOCX]

**Supplementary Table 1 Association between preterm birth and Natal related outcome**

| **Internal association of Preterm birth with lowering APGAR Score, LBW, nursery stay** | | | | |
| --- | --- | --- | --- | --- |
| **PTB** |  |  |  |  |
|  | APGAR | 0·6532 | 0·6711 | 0·1178 to 3·8233 |
|  | LBW | <0·0001 | 18·4026 | 5·1966 to 65·1686 |
|  | NICU | 0·1027 | 2·792 | 0·8136 to 9·5810 |
|  | PPROM | 0·998 | 4·04E+09 |  |
|  | PROM | 0·9991 | 1·09E-09 |  |
|  |  |  |  |  |
| **APGAR** | PTB | 0·9134 | 0·9135 | 0·1788 to 4·6667 |
|  | NICU | <0·0001 | 108·9411 | 12·5967 to 942·1653 |
|  | LBW | 0·1577 | 3·6753 | 0·6041 to 22·3614 |
