# Supplementary material for: Multiple sexually transmitted co-infections are associated with adverse reproductive outcomes in asymptomatic adolescent pregnant women; A Prospective cohort study
Source: Front Med (Lausanne). 2022 Nov 17;9:1046233. doi: 10.3389/fmed.2022.1046233 (PMC9714506; doi:10.3389/fmed.2022.1046233)
Supplement: Supplementary file 2 [file Table_2.docx]

**Supplementary table 2: Distribution of Adverse Outcome in PCR tested negative (control) group and PCR tested positive group**

| **Adverse Outcome** | **Total (279)** | **PCR tested positive (178)** | **PCR tested negative (101)** |
| --- | --- | --- | --- |
| **PTLP** | 32(11.46%) | 28 (15. 73%) | 4 (3.96%) |
| **PTB** | 33 (11.83%) | 28 (15.73%) | 5 (4.95%) |
| **PROM** | 7 (2.51%) | 6 (3.37%) | 1(0.99%) |
| **PPROM** | 6 (2.15%) | 6 (3.37%) | 0 (0.00%) |
| **Leaking per vaginum** | 24 (8.60%) | 18 (10.11%) | 6 (5.94%) |
| **Chorioamnionitis** | 0 | 0 | 0 |
| **LBW (2.1-2.5kg)** | 72 (25.81%) | 57 (32.02%) | 15 (14.85%) |
| **VLBW** | 20 (7.17%) | 18 (10.11%) | 2 (1.98%) |
| **APGAR below 5** | 12(4.30%) | 12 (6.74%) | 0 (0.00%) |
| **NICU** | 28(10.03%) | 23 (12.92%) | 5 (5.00%) |
| **Still birth** | 0 | 0 | 0 |
| **Infant death** | 6 (2.15%) | 5(2.80%) | 1(0.99%) |
